# Enhancing History-Taking Skills in Medical Students: A Practical Guide

**DOI:** 10.7759/cureus.41861

**Published:** 2023-07-14

**Authors:** Reece Patel

**Affiliations:** 1 School of Clinical Medicine, University of Cambridge, Cambridge, GBR

**Keywords:** evidence-based education, medical school exams, osce, medical students, history taking, medical teaching, medical education

## Abstract

History-taking skills are an essential part of the medical school curriculum. However, from the author’s experience and available literature, students consistently report a lack of confidence in their history-taking abilities, and as a result, feel underprepared for upcoming summative communication skills assessments. Therefore, as medical students at the University of Cambridge, we used evidence-based education to create a history-taking teaching programme that aimed to increase confidence and preparedness for communication skills exams. This article outlines 12 tips to be used when teaching history-taking skills to medical students. These tips were created using student feedback, personal experience, and wider literature on the topic. Creating and teaching an effective history-taking course can take significant effort and time. However, we hope this guide provides a starting framework that can be quickly applied to allow users to create effective history-taking skills courses for their students.

## Introduction

There is often an abrupt transition from the preclinical to the clinical course at the University of Cambridge. This is similar across many medical curricula in the UK [1]. Thus, students suddenly undertake clinical placements where they are expected to take histories and examine patients with little preparation or formal instruction. Most students find this a daunting task [2,3]. Incoming clinical students consistently report that they lack confidence in their history-taking ability [4] and feel underprepared for upcoming summative communication skills assessments.

The ability of medical schools to deliver additional teaching is limited pragmatically by two major constraints that are ubiquitous among all medical schools. Firstly, economic factors whereby training students in communication skills is a time-consuming process, particularly when giving one-to-one teaching and feedback. This limits the number of targeted teaching sessions that can be organised and delivered by the clinical school or at teaching hospitals. Secondly, from an administrative perspective, there is significant variation in the number, content, and quality of teaching sessions that students receive between different hospitals. Lack of standardisation of teaching curricula across hospitals and placements will inevitably result in differences in communication skills between students.

Therefore, we used evidence-based education to establish a teaching programme that complemented locally delivered teaching aimed to increase the confidence of medical students in clinical history taking and preparation for summative final assessments. This technical report outlines 12 evidence-based ‘tips’ that should be implemented when medical schools are creating a history-taking skills course.

The technical report is aimed at medical schools looking to implement further history-taking skills teaching. It can also be used by doctors or senior medical students looking to teach history-taking skills to students or their peers.

## Technical report

Tip 1: create cases that are representative of summative assessments

To provide a teaching programme on history-taking skills that is useful and improves preparedness for exams, the cases must be representative of topics that may come up in summative assessments. Medical schools often provide a broad curriculum, which suggests the level of detail at which different conditions should be known. Publicly available summative exam information sheets should be studied to identify common/recurring themes/skills that the students are expected to demonstrate.

Once the cases are written, they should be reviewed periodically by examiners within the clinical school to ensure they are representative and useful for the students [5]. The experts must ensure that the difficulty is fair, the timing allowed is sufficient, and that the case has enough detail to allow all aspects of the clinical history (e.g., social history) to be explored.

Tip 2: involve clinical students/recently graduated doctors in teaching

As explained previously, there may be barriers to increasing the number of university-run history-taking sessions due to financial and administrative reasons. However, this may be partly resolved by asking medical students/recently graduated doctors to volunteer as teachers in the sessions. This would attract enthusiastic teachers looking to increase their teaching experience. This would reduce the cost of the course to the university and increase the number of sessions available to students.

The use of clinical students as teachers has also been shown to improve learning. It has been suggested that learning from near-peers was beneficial because the student teachers had recent experience with the materials and could better appreciate the struggles of being at medical school. However, this also benefitted the student teachers as they could consolidate their knowledge, which is useful for their overall progression [6]. Anecdotally, running the history-taking course benefited the student teachers' preparation for their summative Objective Structured Clinical Examination (OSCE).

Tip 3: ensure the students are aware of the relevance of the course to their exams

The relevance of teaching is the perceived usefulness of the course for the student’s degree, future work, or learning needs. The relevance of learning has been described as being closely linked to the motivation of students to learn [7]. It has been found that higher levels of motivation are associated with the quality of academic behaviours, which could lead to increased achievement [8]. Therefore, we recommend that the relevance of the history-taking course to students’ upcoming exams should be made clear early on. This would aim to increase students' motivation for the course and so improve their learning [9].

Tip 4: the medical history should be taken under exam conditions

Practising taking histories from patients in hospital wards is useful; however, it is often not representative of exam history-taking stations. This is because patients often already know their diagnosis and investigations that have been done in the hospital, and very often tell the student this during the consultation, rather than their initial symptoms. Anecdotally, these practice consultations often take well over the 10 minutes you would usually get in an OSCE. Therefore, although it is essential for medical students to take histories from patients on wards, it is also important that they practice taking structured histories in timed conditions. There are studies to suggest that a good way to learn interview skills is from specific workshops like the teaching course we are proposing [10].

The social constructivism theory [11] applies to teaching history taking. This would suggest that learners would learn best doing a task in the place they would usually do that task [12]. This suggests that medical students would benefit most when taking histories from patients in hospital wards. This has been reported as being beneficial by many Cambridge medical students; however, preparation for summative exams would require practice under exam conditions and so we suggest allowing the students to practice under exam/timed conditions in this course. We suggest running this course alongside students' placements so that they can gain the benefits of both talking to patients and also preparing specifically for summative exams. They can complement each other, allowing students to apply the history-taking techniques they learn in the class, on the wards.

Tip 5: provide an opportunity to practice a structured viva/probe

In medical school, a structured viva/probe often follows a clinical history OSCE station. These are specific questions asked to the student, which are designed to test the student’s clinical reasoning and knowledge of a topic. Using viva/probe questions at the end of the history-taking sessions is useful to students for a number of reasons:

(1) It is a form of retrieval practice. Retrieval practice is ‘bringing learned information to mind from long-term memory’ [13]. There have been a number of papers published supporting the use of retrieval practice over the last 100 years. One such paper has shown that long-term retention is increased by the use of retrieval practice over the use of restudy [14].

(2) By including ‘how’ and ‘why’ questions in the viva, it would also allow ‘elaboration’, another effective learning strategy [13]. Asking the students, ‘why’ or ‘how’ questions around a topic required them to think more deeply about the topic to provide an answer. This is thought to increase knowledge retainment as it integrates the new information with knowledge already stored in memory [13]. After the questions, it is important that their answers are checked and then feedback is given. This is vital because if the answers generated by the student are of low quality, without feedback on the correct answers, it may be detrimental to learning [15].

(3) Practicing answering questions in timed conditions to an examiner would be beneficial to learning due to the social constructivism learning theory [11]. Put simply, this would support the idea that learning is best when the learner is doing the task, in the place where they would do it.

Tip 6: students acting as the patient

Role-play is an important and well-known method of teaching history-taking skills. It draws on an important educational theory: experiential learning. There are four described ‘learning environments’ in experiential learning [16]: (1) concrete experience (feeling); (2) observations and reflections (watching); (3) formation of abstract concepts and generalisations (thinking); (4) testing concepts in new situations (doing).

Structured role-play allows students to complete all of these steps. Although it is important for them to act as a medical professional, this should not be the only role they take on. For students to fully appreciate the doctor-patient interaction, they should also take on the role of the patient. Anecdotally, students reported that after acting as the patient, they better understood how certain questions made them feel, and why specific ways of asking similar questions produce different responses from the patient.

The learning benefit of peer-to-peer role-play, over a patient actor, is contentious with some papers suggesting no difference in communication skills acquired [17]. However, others have found that peer-to-peer role play improved skills more than patient actors with a moderate size effect (Cohen's d = 0.71) [18]. A systematic review has found that student feedback on peer-to-peer role play is globally good in all studies and students reported feeling closer to the patients than in patient actors [19]. Therefore, although the evidence is contentious, there is significant evidence for the use of peer-to-peer role-play.

Although the learning benefit of peer-to-peer role-play is contentious, there is significant evidence that it is less costly than patient actors. One paper found that the global costs for patient actors were five times higher than for peer-to-peer role-play [20]. Another study found that the cost-effectiveness ratio was better for peer-to-peer role-play than for patient actors (0.74 vs. 0.45) [18]. These lower costs would allow for more sessions to be offered to students, which has the benefit of allowing for more history-taking skills practice.

Tip 7: structured feedback is vital

Within medical education, feedback has been described as ‘specific information about the comparison between a trainee’s observed performance and a standard, given with the intent to improve the trainee’s performance’ [21]. Feedback is a vital part of learning [22] and it is an important interaction between the teacher and student.

When providing feedback, we suggest using a structured simplistic mark scheme that the student can look at whilst the teacher explains what was done well/could be improved. This uses the modality principle [23] of cognitive load theory. It is a multimodal approach, which uses both the spoken word and a visual source of information. This aims to reduce extraneous load and so improves learning. It is also important to be selective with feedback and not to bombard the students with too much feedback in one go, as this could leave the student feeling overwhelmed and increase their cognitive load.

Tip 8: ensure that you involve the observing students

It is important to involve the other students in the class (observers) so that they remain attentive and learn from each other. One way to do this is to ask the observers to provide constructive feedback to their peers. Students find providing feedback to peers as beneficial to ‘knowledge and skills development, and the development of professionalism attributes’ [21]. Another option is that you can ask observers to take part in the marking of a peer's history. Peer assessment scores have also been found to be very similar to teacher assessment scores in a meta-analysis of papers (Cohen’s d = 1.91, a high effect size indicates high similarity) [24]. This means, without detriment to the marking, you could ask peers to grade the medical history (using a mark scheme), which may help them familiarise themselves with what examiners are looking for and what are important questions to ask in specific histories. Table 1 represents an example of a structured mark scheme, which was created for a history on abdominal pain. It can be used as a basis to create mark schemes for other cases exploring different systems.

**Table 1:** An example of a structured mark scheme. An example of a structured mark scheme for an abdominal pain history. It also includes a mark scheme for a structured viva after the history has been taken. (1) indicates how the marks are distributed for each statement in the mark scheme. To obtain two marks per section, each (1) statement must be achieved.

Skill & content covered	Marks		
	0	1	2
Introduces themselves (1 = full name and role), and confirm the patient’s name (1)			
Explains the purpose of the interview and obtains consent			
Starts with an open question (1) and allows the patient to speak without interrupting (1)			
Presenting complaint			
Establishes the character (1) and location of the pain including any radiation (1)			
Establishes the onset (1) and the duration (1) of the pain			
Establishes the severity of the pain (1) and whether the patient has taken anything to relieve it (1)			
Asks about associated symptoms such as a change in bowel habits, appetite, nausea/vomiting, urinary symptoms, reflux, etc. (at least 3 = 2, 2 symptoms = 1)			
Enquires whether anything causes the pain to worsen (1) or improve (1)			
Enquires about any similar episodes previously			
Explores the patient’s ideas of the presentation (1) and expectations of the consultation (1)			
Another relevant history			
Systemic features: weight change, appetite, fever, rigours, night sweats (at least 3 = 2, 2 symptoms = 1)			
Relevant past medical (1) and surgical history (1)			
Drug history and allergies			
Relevant family history			
Smoking (including ex-smoker) and alcohol use			
Relevant social history (e.g., diet, exercise/baseline mobility, children, and occupation)			
Appropriate closure of consultation (e.g., explaining next step), summarises and thanks the patient			
Communication skills			
Appropriate questioning style (starting off open and becoming more focused)			
Attends appropriately to patient's questions (1), listens actively (1)			
History taking is done in an organised manner			
Viva			
What is your list of differentials?			
What is the most likely diagnosis and why?			
How would you investigate this patient?			

Tip 9: encourage the student to reflect

Reflection is ‘a metacognitive process that creates a greater understanding of both the self and the situation so that future actions can be informed by this understanding’ [25]. In a systematic review, evidence showed that reflection was associated with ‘deeper learning’ and this improves integration between new learning and existing knowledge [26]. As described previously, Kolb and Fry (1974) outlined the four stages of experiential learning, the second stage of which requires reflection, where the learner attempts to understand what occurred in the experience, and how they reacted. During this stage, they can identify further learning needs, such as knowledge or skills needed. This can then be applied during the fourth stage (testing concepts in new situations). If this cycle is repeated multiple times, it can help to increase learning [25], consequently enhancing the learning experience for medical students.

We recommend the use of guided reflection, with the teacher facilitating the student’s reflection. This is useful as the student may not always notice gaps in their knowledge or things they did well. A teacher can support the student in noticing and making sense of different sections of the history-taking experience, to facilitate effective reflection. In a review of the evidence, students appreciated help from a supervisor to ‘facilitate their reflection’ [25]. Therefore, we recommend guided reflection for each student after they take a history.

Tip 10: use appropriate learning outcomes and include a quiz

There are many benefits of well-written learning outcomes, including reducing stress on students and providing students with a list of exactly what they should know from each session [27]. They should be written at the start of the session and then brought up again at the end of the session so it is clear how each learning objective has been met.

We recommend using a short, informal quiz at the end of the session to improve the retainment of information from the session through retrieval practice. The quiz could test things like important questions to ask in the history of certain body systems, or how to ask specific questions. It should cover important themes that were brought up in the session.

Tip 11: teacher to be enthusiastic and friendly

Evidence shows that enthusiastic and friendly teachers consistently increase intrinsic motivation in students, increasing interest and making educational sessions appear shorter [28]. A small meta-analysis of the data has shown that teachers' enthusiasm for a subject has increased educational performance in higher education with a medium effect size (Cohen’s d = 0.56) [29]. Therefore, enthusiastic and friendly teachers not only increase motivation but also improve educational performance. Anecdotally, enthusiastic and friendly environments often make students feel more comfortable and so we have found participation and reflection more useful in these environments.

Tip 12: gain feedback from the students

Student feedback informs the teachers of their strengths and weaknesses [30] and allows them to identify areas for professional development. A student feedback form at the end of the session allows students to voice what they enjoyed/found useful about each session and also what could be improved. It also allows them to provide what specific history-taking content they would like the teachers to cover, which can help tailor the teaching to the student’s needs. In one study looking at student feedback in medical education, over two-thirds of students and teachers agreed that ‘student’s feedback is an effective tool for the faculty development’ and that it sensitized teachers towards the student's needs [30]. Therefore, we recommend teachers gather anonymous student feedback after each session, reflect on the feedback and use it to guide the development of future sessions.

## Discussion

History-taking skills are an important part of the medical school curriculum. However, medical students consistently report a lack of confidence and under-preparedness for communication skills assessments. The ability of medical schools to deliver additional teaching is often limited due to economic and time constraints. Therefore, creating an effective history-taking course is difficult and requires planning. In this article, we have outlined 12 tips that can be implemented when creating a history-taking course. These 12 tips have been summarised in Table 2.

**Table 2:** A summary of the 12 tips included in this article and the important takeaway points from each tip.

Tips	Important takeaway points
Tip 1: Create cases that are representative of summative assessments	The cases used in a history-taking programme should be representative of topics that may come up in assessments
Tip 2: Involve clinical students/recently graduated doctors in teaching	Learning from near-peers can be beneficial to learning
	This could reduce costs and increase the number of sessions available to students
Tip 3: Ensure the students are aware of the relevance of the course to their exams	Explaining the relevance of the course can help to improve motivation and may increase achievement
Tip 4: The medical history should be taken under exam conditions	Learning is best when the learner is doing the task, in the place they would do it
Tip 5: Provide an opportunity to practice a structured viva/probe	Is a good way of incorporating retrieval practice into sessions
	Allows for the use of elaboration, an effective learning style
	Learning is best when the learner is doing the task, in the place they would do it
Tip 6: Students acting as the patient	Structured role-play allows students to complete all of the steps of experiential learning
	Can reduce the cost of the history-taking programme
Tip 7: Structured feedback is vital	Use a structured simplistic mark scheme that the student can look at whilst the teacher explains what was done well/could be improved
Tip 8: Ensure that you involve the observing students	Ensures the observers remain attentive and learn from each other
	Students find providing feedback to peers beneficial
Tip 9: Encourage the student to reflect	Reflection is associated with improved learning and integration between new knowledge and existing knowledge
Tip 10: Use appropriate learning outcomes and include a quiz	Effective learning outcomes can reduce the stress of students
	Learning outcomes should be written at the start of the session and then brought up again at the end of the session
	A quiz can help learning, as it uses retrieval practice
Tip 11: Teacher to be enthusiastic and friendly	This increases motivation, student interest, and participation and makes educational sessions appear shorter
Tip 12: Gain feedback from the students	Student feedback informs the teachers of their strengths and weaknesses and is an effective tool for faculty development

The use of evidence-based education techniques will help to ensure that history-taking skills teaching is useful to medical students and aids their learning. The tips we have suggested are supported by evidence and give guidance on not solely what to teach, but also how to teach it. Ensuring the teaching is done under exam conditions and giving structured feedback have been shown to improve learning [10,21]. However, learning is also improved by simple behaviours like showing enthusiasm and being friendly [28,29]. Therefore, although it may not be practical to immediately apply all the tips we have suggested, making simple changes could help to improve history-taking skills and students’ confidence for final exams.

One theme that has appeared consistently throughout this article is the importance of feedback. Feedback is a vital part of learning and should be provided not only by the teacher but also by the student's peers. It has been found that providing feedback to peers is beneficial for knowledge and skills development as well as professional attributes [21]. It has also been shown that feedback provided by students to the teacher on the quality of the teaching session may be useful in guiding them and allowing them to tailor content to a particular cohort [30]. Therefore, feedback is not only important to the students, but it is also useful for the teachers, as it enables them to improve the teaching sessions. Feedback is also useful to allow effective reflection by students. Students may not always notice areas for improvement on their own. However, they appreciated help from their teachers to facilitate effective reflection [25] by noticing knowledge gaps or things the student had done well. Therefore, feedback and guided reflection should be used after each student's history-taking role-play.

We have found that it is essential that the history-taking teaching programme is representative and relevant to students’ communication skills exams. The motivation of students is closely linked to the relevance of learning [7], and it has been shown that increased motivation is associated with behaviours, which may lead to increased achievement [8]. Therefore, the course should be relevant to the students’ exams, and this should be made clear to them from the start of the course. Taking the histories under exam conditions and using a structured viva/probe help to ensure the history-taking teaching programme is representative of communication skills exams. The social constructivism learning theory would suggest that learning is best when the learner is doing the task, in the place they would do it. Therefore, preparation for summative exams should be done under exam conditions, with a structured viva at the end as this simulates a communication skills station in an exam.

The tips suggested can be used to improve learning, but also reduce cost and so allow for more history-taking sessions for students. Tip 2 suggested we could ask medical students/recently graduated doctors to volunteer as teachers. This is beneficial to both the course developer (teaching is cost-free) and also to the volunteer teachers, as they will gain teaching experience as well as help them to consolidate their knowledge and learning [6]. Another way we have suggested that costs can be reduced is by using peer-to-peer role-play (outlined in tip 6). It has been calculated that the global costs of a patient actor are five times that of peer-to-peer role-play [20] and that the cost-effectiveness ratio was better for peer-to-peer role-play than for patient actors (0.74 vs. 0.45) [18]. Therefore, by using peer-to-peer role-play, the costs of teaching can be significantly reduced.

To the author’s knowledge, there are no published guidelines on how to teach history-taking skills. Our review gives a set of evidence-based tips that can be applied when creating a history-taking skills programme. As our understanding of learning progresses and new teaching techniques are developed, these tips could be expanded upon in the future.

## Conclusions

History-taking skills are an essential component of clinical competence, forming the cornerstone of safe and effective clinical practice. For this reason, evidence-based education should be applied to ensure it is taught in the most effective way to produce the best clinical communicators. These 12 tips provide a good baseline framework for how to apply evidence-based education to history-taking teaching. However, we recommend readers continue to find evidence in this area and apply it to their teaching practices. From experience, creating and teaching a history-taking course can take significant effort and time. However, we hope this guide provides a starting framework so this process can be streamlined and students can quickly gain the benefits of the techniques we have outlined.
